# An update on the CHDGKB for the systematic understanding of risk factors associated with non-syndromic congenital heart disease

**DOI:** 10.1016/j.csbj.2021.10.017

**Published:** 2021-10-13

**Authors:** Lan Yang, Xingyun Liu, Yalan Chen, Bairong Shen

**Affiliations:** a Center of Prenatal Diagnosis, Wuxi Maternal and Child Health Hospital affiliated to Nanjing Medical University, Wuxi, China; b Center for Systems Biology, Soochow University, Suzhou 215006, China; c Institutes for Systems Genetics, Frontiers Science Center for Disease-related Molecular Network, West China Hospital, Sichuan University, Chengdu, Sichuan 610041, China

**Keywords:** Congenital heart disease, Risk factor, Environmental, Non-syndromic

## Abstract

The Congenital Heart Disease Genetic Knowledge Base (CHDGKB) was established in 2020 to provide comprehensive knowledge about the genetics and pathogenesis of non-syndromic CHD (NS-CHD). In addition to the genetic causes of NS-CHD, environmental factors such as maternal drug use and gene-environment interactions can also lead to CHD. There is a need to integrate this information into a platform for clinicians and researchers to better understand the overall risk factors associated with NS-CHD. The updated CHDGKB contains the genetic and non-genetic risk factors from over 4200 records from PubMed that was manually curated to include the information associated with NS-CHD. The current version of CHDGKB, named CHD-RF-KB (KnowledgeBase for non-syndromic Congenital Heart Disease-associated Risk Factors), is an important tool that allows users to evaluate the recurrence risk and prognosis of NS-CHD, to guide treatment and highlight the precautions of NS-CHD. In this update, we performed extensive functional analyses of the genetic and non-genetic risk information in CHD-RF-KB. These data can be used to systematically understand the heterogeneous relationship between risk factors and NS-CHD phenotypes.

## 1 Introduction

Congenital heart disease (CHD) is the most common cause of heart disease with an estimated incidence of 0.7–1% per live birth [1], [2]. Reports have shown that genetics plays an important role in the process of CHD and that chromosomal abnormalities, copy number variations, mutations (including single nucleotide polymorphism) [3], [4], [5], hypomethylation [6] and functional variants in microRNAs contribute to the development of CHD [7]. These genetic variations disrupt or alter the function of genes during the normal development of the heart. Whilst genetics play a vital role in the development of CHD, only 20–30% of individuals with CHD can be identified based on a single genetic factor [8]. Large-scale studies have suggested that environmental factors such as parental drug profiles, maternal health status can cause or interact with genetic variations to contribute to CHD [9], [10], [11], [12].

Advances in genetic testing and surgical techniques have led to a decrease in the prevalence of CHD. However, there are currently no available comprehensive risk factors for the genetic and non-genetic information associated with NS-CHD. Syndromic CHD describes CHD with syndrome-associated abnormalities such as Noonan, DiGeorge, Holt-Oram, Marfan, Chat and other syndromes, often with cardiac and non-cardiac abnormalities. Non-syndromic CHD refers to CHD with only cardiac abnormalities including simple and severe congenital heart disease.

The current version of CHDGKB was developed from articles available on PubMed. We estbalished a genetic variation database and included an analysis of the molecular mechanism of NS-CHD. The updated database presented in this study provides a useful tool for researchers to systematically study the prognosis, risk of recurrence, and to evaluate treatments for NS-CHD. Also, in the current version, we performed extensive functional analyses aiming to better understand the complex relationships between genes, NS-CHD subtypes, and other risk factors.

## 2 Data collection and knowledgebase structure

### 2.1 Data collection

Based on the CHDGKB database, we expanded all of the non-genetic risk information associated with NS-CHD. In the updated version, we collected all data for the KnowledgeBase on non-syndromic Congenital Heart Disease associated Risk Factors (CHD-RF-KB) manually from PubMed. The literature searches were performed on publications prior to May 5th, 2020 with the following keywords were included: (congenital heart disease[title]) AND (biomarker*[title] OR marker*[title] OR indicat*[title] OR predict*[title] OR associat*[title] OR risk factor*[title/abstract] OR risk model*[title/abstract]). 386 out of 1,517 publications from 1998 to 2020 were selected for the updated NS-CHD risk factor database.

### 2.2 Inclusion and exclusion criteria

The inclusion criteria for the non-genetic risk data in the CHD-RF-KB were partly the same as that for the CHDGKB [13]. The studies had to meet the following criteria: 1) Patients presented with the clinical features of CHD and had echocardiographic evidence of disease or surgical records; 2) Studies conformed to approved institutional guidelines and all patients were recruited by written informed consent; 3) Patients had established environmental risk factors for CHD including maternal illnesses, drug use during the first trimester of pregnancy, parental smoking, and chronic exposure to toxic substances or ionizing radiation.

The exclusion criteria for the non-genetic risk data were the same as those for the CHDGKB [13] criteria (i), (ii), (iii).

### 2.3 Database construction

**Fig. 1:**
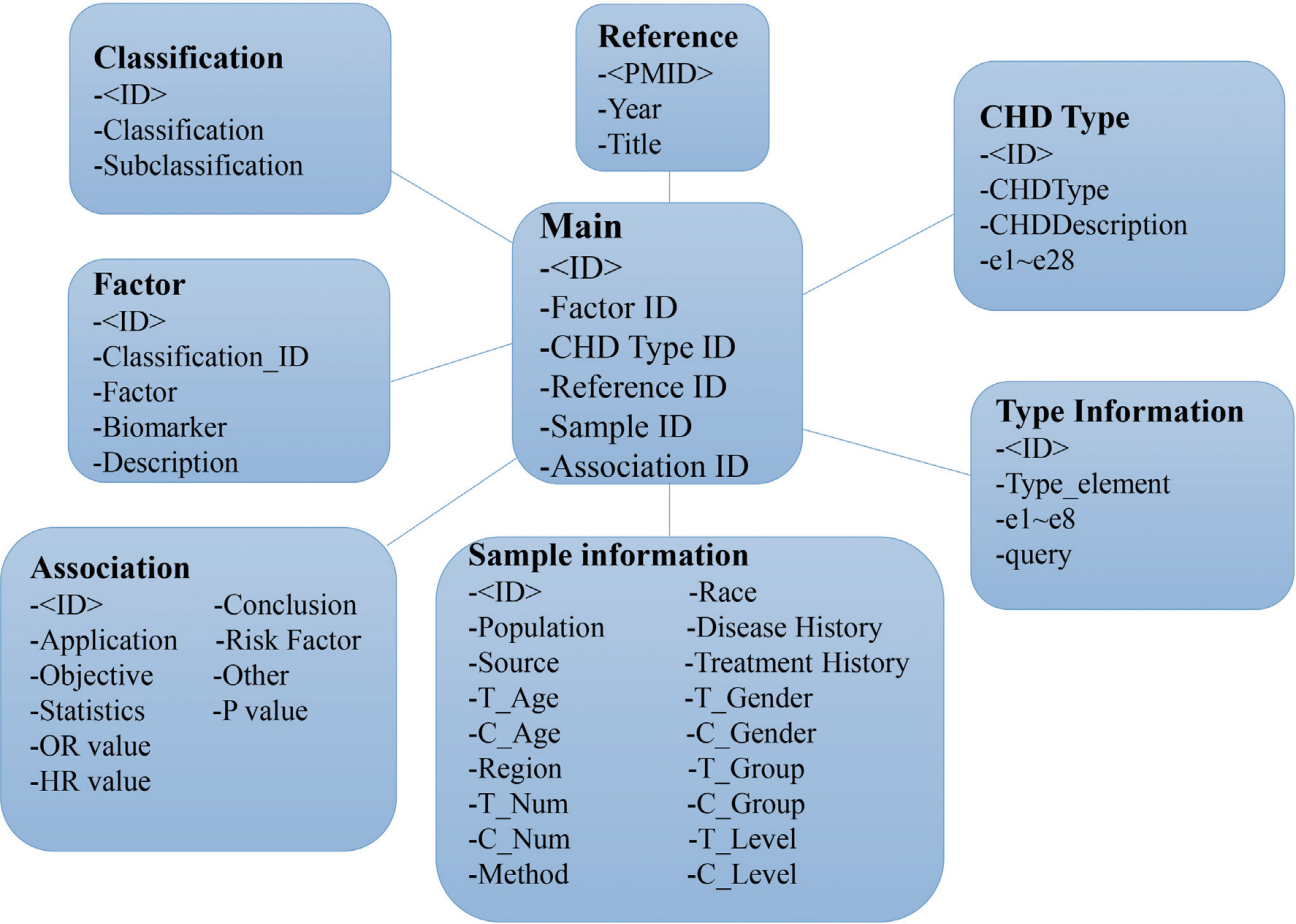
The UML (Unified Modeling Language) diagram of the CHD-RF-KB with non-genetic risk factors.

The CHD-RF-KB web interface was constructed with MySQL (10.4.6-MariaDB), Apache (2.4.39), PHP (7.3.8), HTML, Bootstrap 4, and JavaScript. An overview of the construction of CHD-RF-KB with non-genetic factors is shown in Fig. 1.

## 3 Update and extension

### 3.1 Updating genetic information

**Fig. 2:**
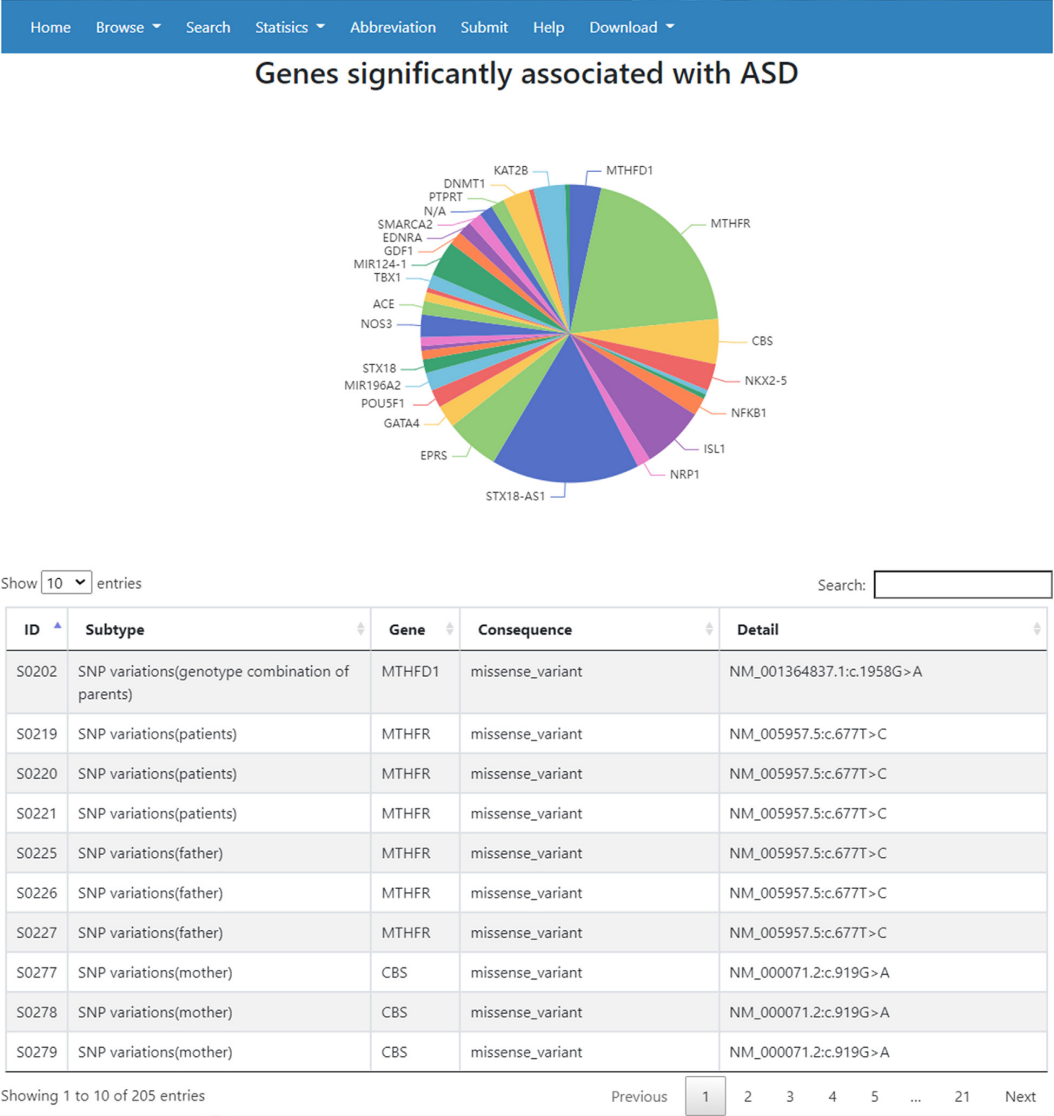
Diagram showing the correlation between ASD and associated genes (the top 20 genes with all variations for ASD are listed).

**Fig. 3:**
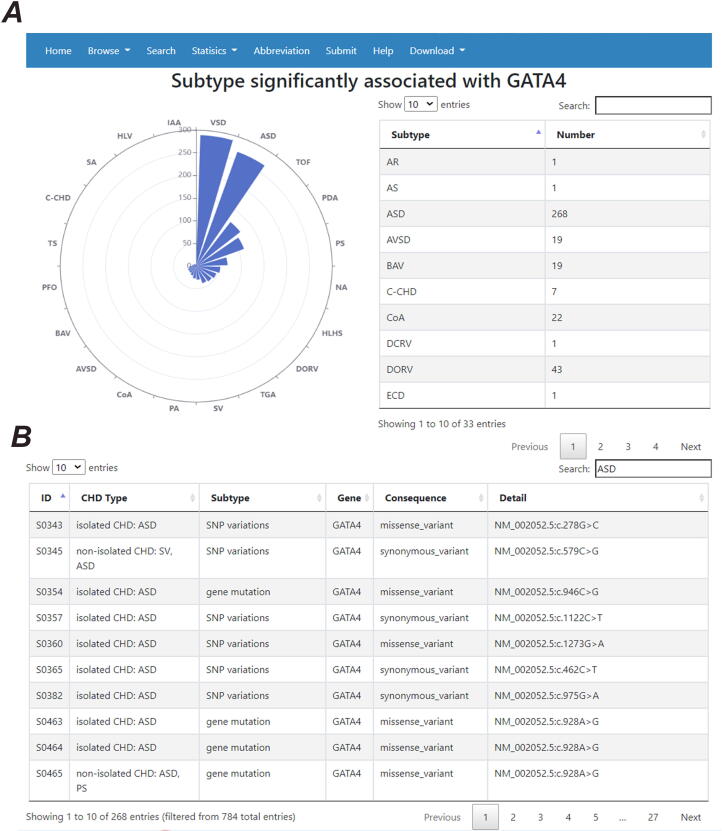
An example of the correlation between genes and the NS-CHD subtype. A: Correlation between GATA4 and associated NS-CHD subtypes; B: Variation data of ASD associated with GATA4. Only the top 20 subtypes with variations for GATA4 are listed.

The updated version of CHDGKB includes the details from 284 individual studies. Up to 5th May 2020, the data from 697 studies were manually mined in the CHD-RF-KB version. The genetic information was updated to include 5521 items consisting of 4830 small variations, 657 copy number variations (CNVs), 17 methylations, and 17 other genetic variations. The small variations included 3714 SNPs, 1057 mutations (NOT SNP), 12 haplotypes, and 47 other variations. In our current version, we also extended the related statistical function between the NS-CHD subtypes and variant genes (correlation criteria: P < 0.05). Taking atrial septal defects (ASDs) as an example, when the input “ASD” was input as the “subtype” on the “Statistics” interface, the webpage can show a correlation diagram between “ASD” and all related genes, as presented in Fig. 2. When the input “GATA4” was input into the “Gene” interface, a correlation diagram between “GATA4” and related subtypes and corresponding genetic information is presented (Fig. 3).

### 3.2 Extension to the non-genetic factors

**Fig. 4:**
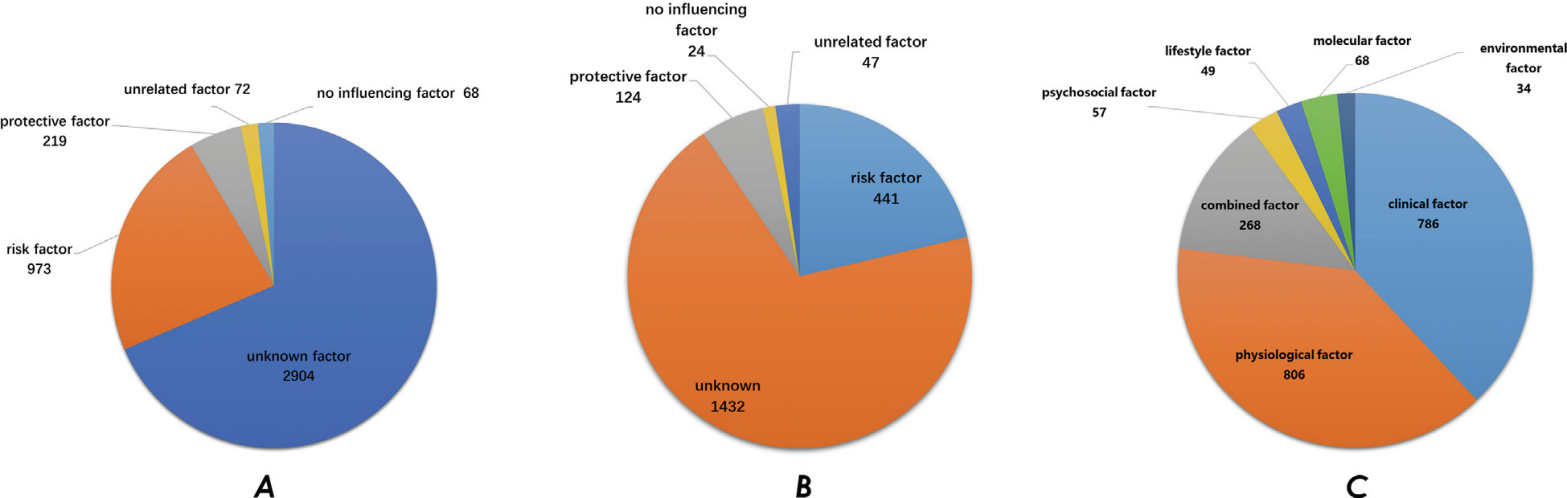
A: The distribution of five classifications with risk factors for NS-CHD; B: The classifications of risk factors associated with ASD; C: The sub-classifications of risk factors associated with ASD.

**Fig. 5:**
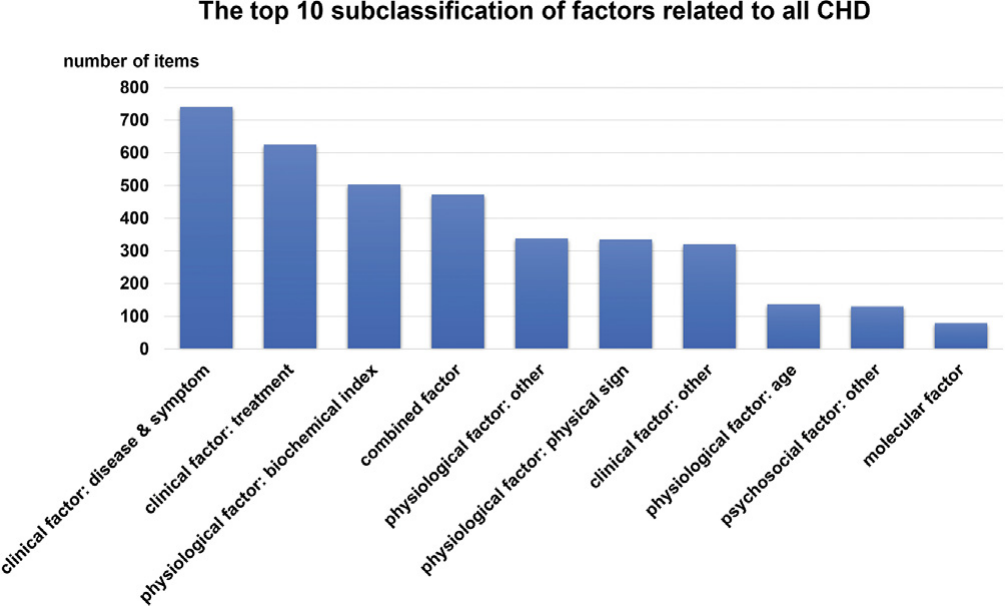
The top 10 sub classifications of non-genetic risk factors for NS-CHD.

An extension to the non-genetic factors associated with NS-CHD in CHD-RF-KB was made. The risk factors were classified into five groups as shown in Table 1 [14], [15]. Based on these definitions, the 4,236 non-genetic risk factors were distributed as risk (23%), protective (5.2%), non-influencing (1.6%), unrelated (1.7%) and unknown factors (68.6%) (Fig. 4A). The non-genetic risk factors were further divided into seven subgroups as clinical (42.3%), environmental (1.0%), lifestyle (2.5%), molecular (2.4%), physiological (36.05%), psychosocial (4.6%) and combined factors (11.14%). Each of the seven sub-classifications had specific details that were sorted according to the top 10 sub-classifications of risk factors that were correlated with all of the NS-CHD subtypes. These data are presented in Fig. 5.

**Table 1:** Definition for the five categories of risk factors.

Risk factor type	P value	Effect index
Protective	p < 0.05	OR(HR) < 1.0
No influencing	p < 0.05	1.0 ≤ OR(HR) < 1.2
Risk	p < 0.05	OR(HR) ≥ 1.2
Unrelated	p < 0.05	–
unknown	Other (except for situations mentioned above).	–

**Fig. 6:**
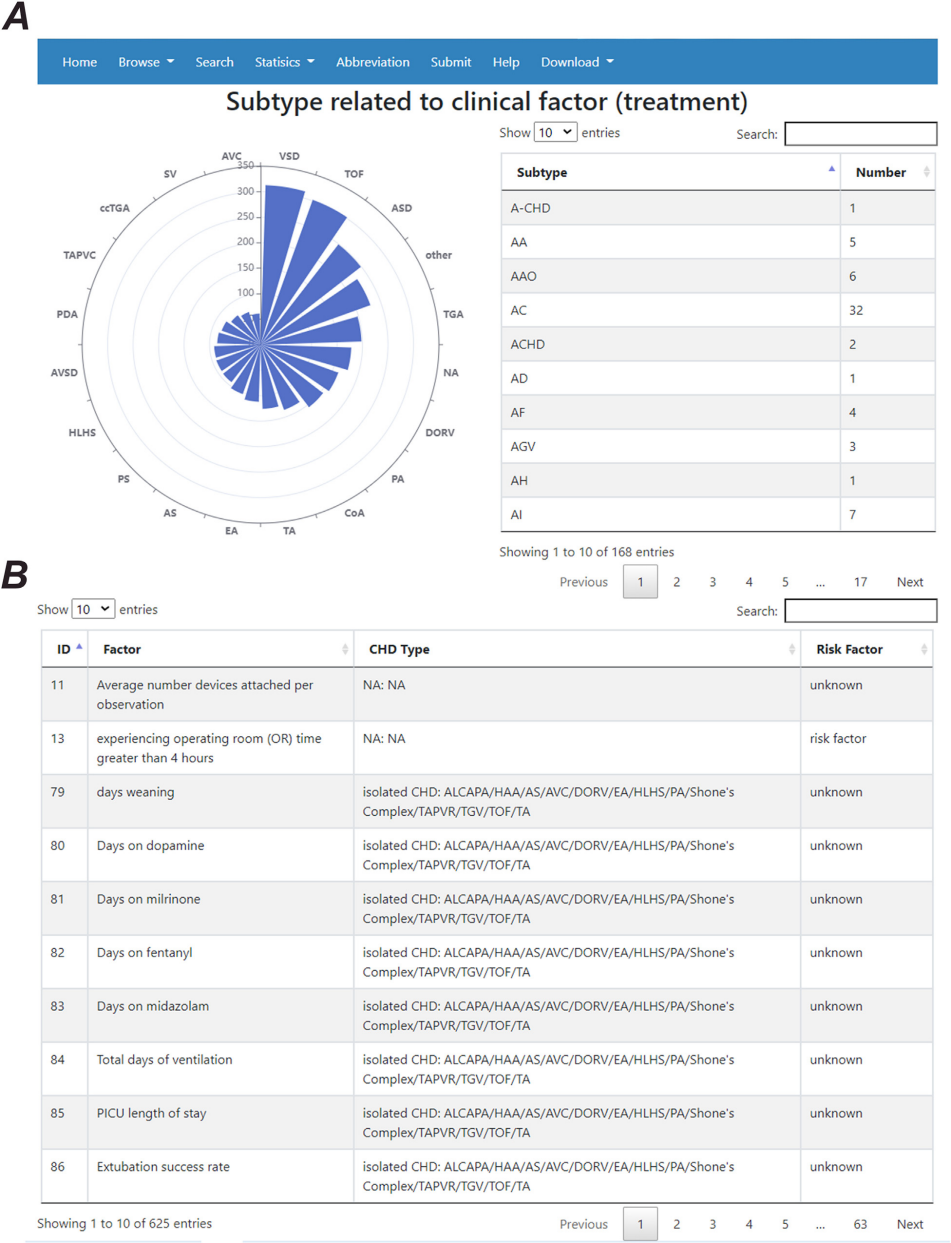
An example correlation diagram between risk factors and the NS-CHD subtype. A: The correlation diagram between treatment and associated NS-CHD subtypes (Only the top 20 subtypes with risk factors for treatment are listed); B: The risk factor data for subtypes associated with treatment.

Similar to the correlation functions at the genetic interface, this new function was extended to the “non-genetic” interface in which users can search for all of the risk factors associated with a certain subtype. For example, when type “ASD” in the “non-genetic” details were entered into the interface, the webpage shows a classification of the risk factors related to ASD (Fig. 4B) along with a sub-classification of the factors associated with ASD (Fig. 4C). Users can search all of the NS-CHD subtypes that are correlated with a specific factor. When the input is a “treatment” in the sub-classification of factor interface, a correlation diagram between the treatment and the associated subtypes is shown in the statistics interface, along with the correlated risk factor information (Fig. 6).

## 4 Data access and exploration

### 4.1 Data browsing and retrieval

Users can browse the risk factor data by choosing the classification, sub-classification, factors and risk factors (e.g. protective or unrelated factors). The users can search for information on the non-genetic risk factors related to a certain CHD subtype on the query interfaces through the following processes:

1. Search the CHD-subtype in the “Contain” menu.
2. Search the “exact” menu: Users can search for any of the NS-CHD types/subtypes by selecting the terms from the drop-down menu which is a precise query.

### 4.2 Data Download and submission

Similar to the CHDGKB version, all of the NS-CHD non-genetic information can be downloaded in Excel format (http://www.sysbio.org.cn/CHDRFKB/Download.html). The risk factor data can be submitted to repositories at http://www.sysbio.org.cn/CHDRFKB through the “Submit” interface for further validation and updating.

### 4.3 Non-genetic risk factors correlated with NS-CHD subtypes

**Fig. 7:**
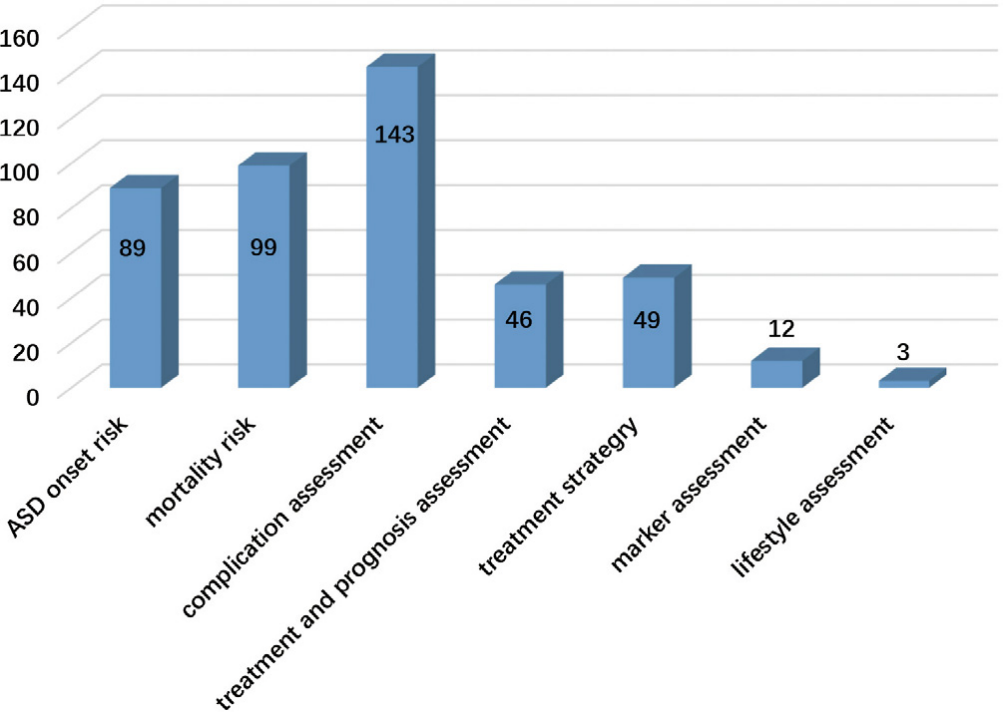
The distribution of risk factors in the application associated with ASD.

ASD was selected as an example which was reported as one of the most common subtypes in the CHD-RF-KB. Five types of risk factors correlated with ASD are shown in Fig. 4B. These risk factors can be separated into those related to ASD risk and those that are correlated with ASD prognosis. The distribution of these factors in the application is shown in Fig. 7. 89 risk factor items aimed at ASD risk based on single factors classification of cardiovascular diseases [16]. These were divided into seven sub-classifications as clinical (30 items), physiological (10 items), molecular (16 items), environmental (9 items), psychosocial (7 items), combined (7 items) and lifestyle factors (4 items).

### 4.4 Genetic risk factors correlated with NS-CHD subtype

**Fig. 8:**
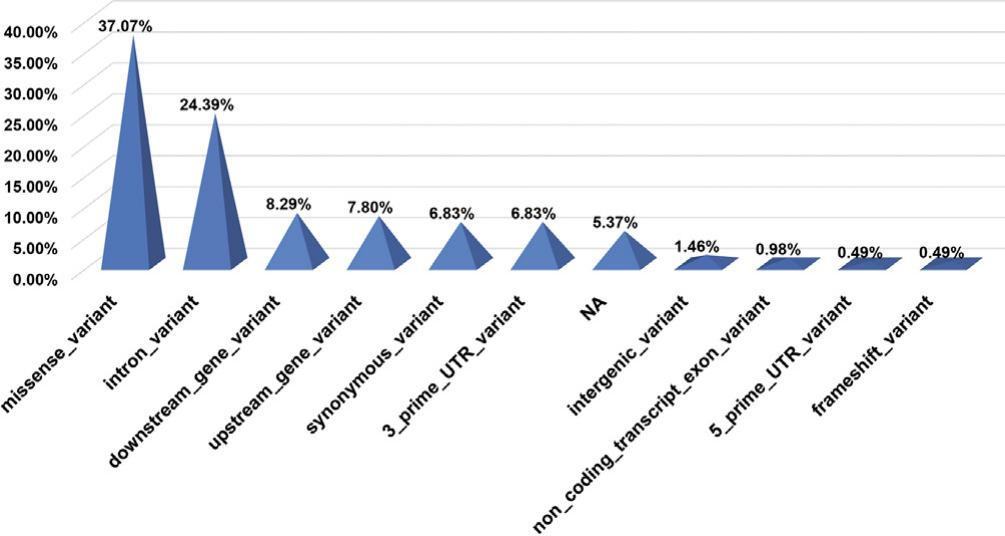
The distribution of genetic variation types associated with ASD.

Using ASD as an example and based on a criterion of *p* < 0.05, when “ASD” was the “genetic” input and the “CHDsubtype” input of web statistic interface, a list of 205 items with genetic variations was shown. Amongst the genetic variations that were correlated with ASD, a total of 31 genes were identified. There were 11 variation types related to ASD that are shown in Fig. 8. Amongst the 11 variation types, missense and intron variants accounted for the top two proportions of the variants at 37.07% (76 items) and 24.39% (50 items), respectively. The remaining 9 variation types included downstream, upstream, synonymous, 3 prime UTR, 5 prime UTR, intergenic, non-coding transcript exon, frameshift and unknown variants.

### 4.5 GO enrichment analysis and pathway mapping

**Fig. 9:**
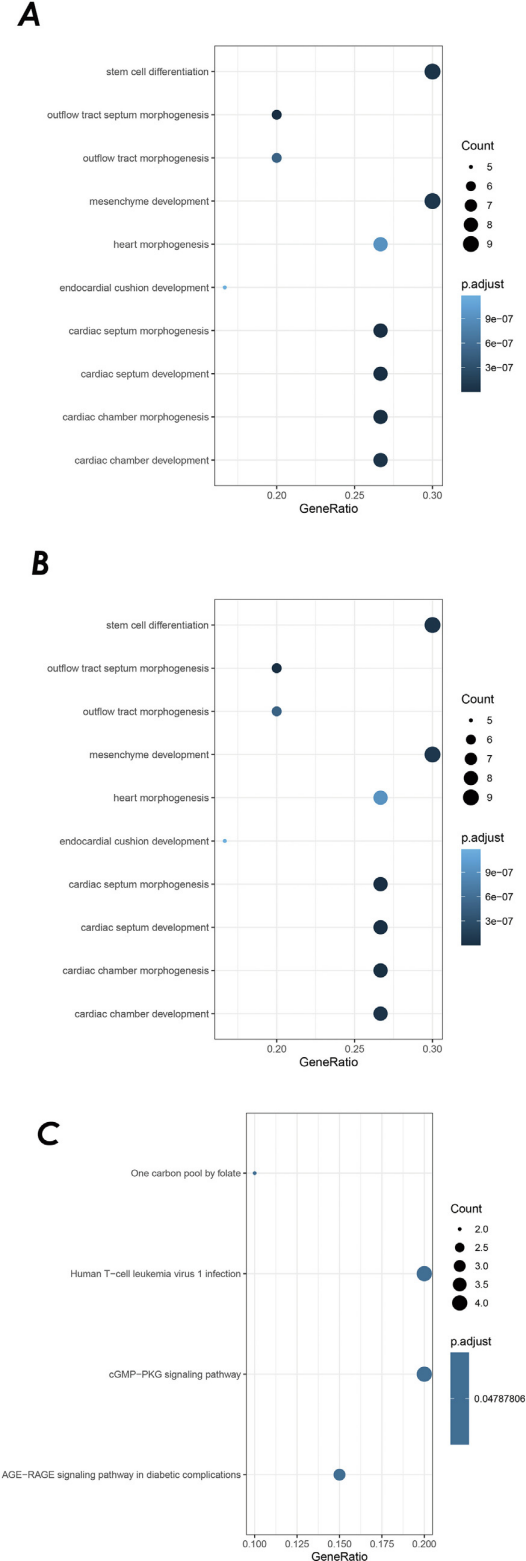
A: The top 10 most significantly enriched GO terms at the BP level with ASD; B: The top 10 most significantly enriched GO terms at the MF level with ASD. （Black dots indicate the number of enriched genes; Y-axis indicated Gene Ontology terms). The statistical significance level (p.adjust, adjusted P-value) is depicted as different colors; C. Pathway enrichment analysis for the genetic variations of ASD. The statistical significance level (p.adjust, adjusted P-value) is depicted as different color. (The top 4 most significant KEGG terms for ASD. Black dots indicate the number of enriched genes; Y-axis indicate the enriched pathways).

The R package ClusterProfiler was used for the GO (Gene Ontology) analysis of the ASD subtype at the biological process (BP), cellular component (CC), and molecular function (MF) levels. The associated genes and the number of enriched GO terms are listed in Table 2. The top 10 significantly enriched terms (*p* < 0.05) on the two process levels for ASD are summarized in Fig. 9A and 9B. For the ASD subtype, at the BP level, the most significantly enriched terms were mainly related to stem cell differentiation, mesenchyme development, cardiac septum morphogenesis/development and cardiac chamber morphogenesis/development.

**Table 2:** The genes and enriched GO term numbers associated with ASD.

Gene_NAME	SEQ_NAME	BP	MF	CC
NRP1	NM_003873.6	285	13	19
ISL1	NM_002202.3	219	14	0
NOS3	NM_000603.5	196	18	7
BMPR1A	NM_004329.2	195	6	8
GATA4	NM_002052.4	166	8	3
NKX2-5	NM_004387.4	154	1	4
NFKB1	NC_000004.12	147	6	5
TBX1	NM_005992.1	127	4	0
MIR138-2	NC_000016.10	120	1	0
TBX2	NM_005994.4	110	5	1
ACE	NM_000789.4	92	14	1
KAT2B	NM_003884.5	87	18	16
DNMT1	NM_001379.4	78	6	5
ZW10	NM_004724.4	67	0	11
MTHFD1	NM_005956.4	65	6	0
MTHFR	NM_005957.5	60	7	0
EDNRA	NC_000004.12	54	8	0
MTRR	NM_002454.3	50	9	0
MIR196A2	NC_000012.12	36	1	0
EPRS	NM_004446.3	30	6	1
CBS	NM_000071.2	29	16	0
NFATC1	NC_000018.10	29	8	3
POU5F1	NC_000006.12	26	5	0
PTPRT	NC_000020.11	19	13	0
STX18	NC_000004.12	13	1	1
FIGN	NC_000002.12	12	3	3
GDF1	NM_001492.6	10	5	0
SMARCA2	NC_000009.12	10	6	7
TLL1	NM_001204760.2	3	3	0
STX18-AS1	NC_000004.12	0	0	0
MIR124-1	NC_000008.11	0	0	0

At the MF level, the most significantly enriched terms were mainly correlated with DNA-binding transcription activator activity, RNA polymerase II-specific, RNA polymerase II transcription factor binding and activating transcription factor binding. KEGG pathways were also generated based on the enrichment analysis. The top four significantly enriched terms of the KEGG pathways for ASD are summarized in Fig. 9C. The cGMP-PKG signaling pathway, Human T-cell leukemia virus 1 infection, AGE-RAGE signaling pathway in diabetic complications, and the one-carbon pool by folate were pathways identified as essential for the occurrence of the ASD subtype.

## 5 Discussion

### 5.1 Correlation analysis of the non-genetic risk factors associated with ASD

**Fig. 10:**
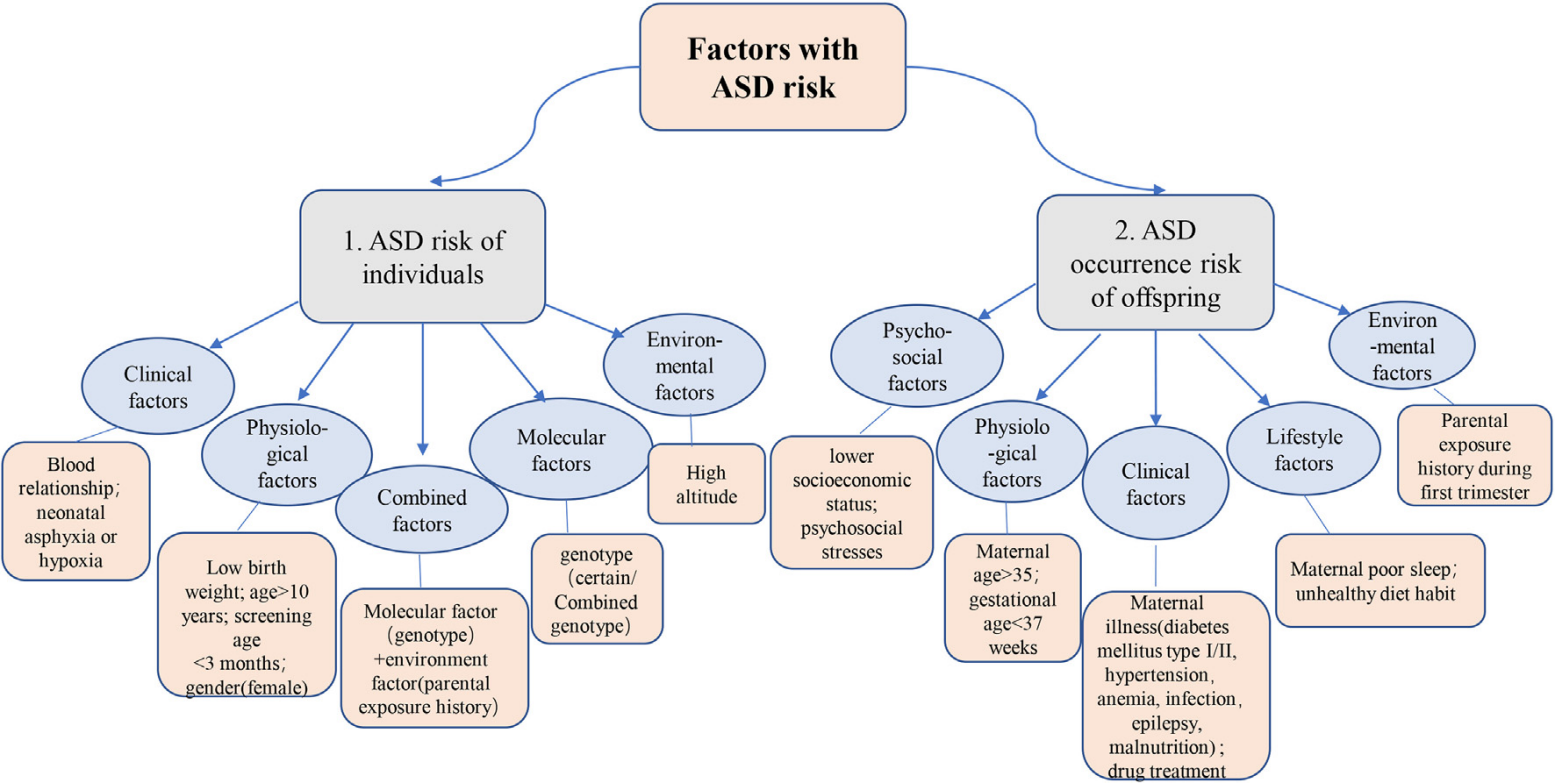
The factors associated with the risk of ASD.

As shown in Fig. 9, the 441 risk factors associated with ASD were correlated with complications, mortality, and ASD occurrence risk. 89 items that were ASD risk factors were selected for further correlation analysis. These risk factors were correlated with the ASD risk of individuals or with the ASD risk in the offspring of the individuals. The detailed risk factors associated with two categories are listed in Fig. 10.

The physiological factors for ASD were mainly birth weight, detection age, and gender. It was suggested that birth weights less than 2500 g, screening at 0–3 months and neonates with asphyxia or hypoxia had a diagnostic risk factor for ASD [17], [18]. Individuals aged 10–40 years and females had a high risk of ASD compared to males aged 0–9 years [19]. Amongst the molecular factors, the genotype of the *MTHFR* gene (c.677C > T: CT or TT) was correlated with ASD [20]. Wang et al [21] reported the prevalence of ASD to be 43% in first-degree relatives which was significantly higher than 4.4% in second-degree relatives. Furthermore, the prevalence of ASD (90%) in twins was significantly higher (62.2%) than in siblings. These data indicate that genetic factors play an important role in the development of CHD.

Amongst the combined factors, it was found that genetic variation combined with harmful parental environments or unhealthy lifestyles were associated with ASD risk. For instance, a functional Aryl hydrocarbon receptor (AhR) genetic variant (p.Arg554Lys) (rs2066853) is a risk factor for ASD alone. Individuals carrying genetic variants of Arg (genotype with Lys/Lys and Arg/Lys) had a parental history of exposure to toxic environments or smoking, and so the risk of ASD was significantly higher than those without exposure histories [12]. Genetic and environmental factors may contribute to the development of CHD. Furthermore, in the fetal order [18], along with ascending altitude environment [22], the prevalence of ASD increased accordingly. Therefore, the age of screening, females, high altitude environments and first or second-degree relatives of CHD patients are at risk.

52 ASD risk factors associated with offspring mainly included maternal diseases, environmental exposures, maternal psychosocial and physiological factors, unhealthy lifestyles, and drug treatments. Firstly, maternal illnesses such as diabetes mellitus (type 1, 2), hypertension before and during pregnancy, anemia, epilepsy, connective tissue disorders, and mood disorders were all identified as risk factors for ASD [23]. Moreover, maternal respiratory tract infections [18], vaginal infections, and clotting disorders were all significantly associated with ASD [24]. Also, pregnancy malnutrition and histories of abnormal childbearing were found to be correlated with ASD in offspring [25]. Maternal illness and diabetes mellitus (type 2) were related to the risk of ASD occurrence in offspring and also increased the severity of CHD [23].

Dolk et al, found that increased paternal blood pressure and the use of anti-clotting medications (enoxaparin and aspirin) in the first three months of pregnancy were correlated with ASD [24]. Therefore, we need to prevent high-risk diseases such as diabetes mellitus before and during pregnancy, especially for those that may involve high blood pressure in both parents. Also, several factors should be prevented including upper respiratory tract infections and the use of medication during early pregnancy. Health education should be offered to women of childbearing age along with the use of improved obstetric procedures and techniques to reduce the risk of CHD.

Medication history and exposure to adverse environmental factors were shown to increase the prevalence of ASD in offspring. For example, exposure to decoration environments during pregnancy increases the risk of isolated CHD such as ASD or VSD in offspring and is significantly correlated with complex CHD. Moreover, exposure to housing renovations in the first trimester (less than one month after renovation) increases the risk of ASD in offspring more than before pregnancy [26]. This may be due to the teratogenic sensitive period of the embryo in the first pregnancy trimester.

In addition, unhealthy maternal lifestyles are related to the occurrence of ASD. Studies have shown that poor maternal sleep can increase the risk for ASD and other CHD subtypes in offspring. Within the same group of pregnant women with poor sleep quality, the concurrence of daytime naps decreases the risk of simple CHD [27]. Dolk’s research also showed that mothers who drink fizzy or high-energy drinks every day had a higher risk of ASD in their offspring [24]. Maternal physiological and psychosocial factors were also correlated with the risk for CHD in the offspring. Mothers over 40 years of age and gestational ages less than 37 weeks were all at higher risk for ASD compared to younger pregnant women and full-term deliveries. Mothers with blue-collar occupations [28], lower education levels, multiple stresses in the periconceptional period, and other social psychology factors also had a higher prevalence of CHD in offspring [24].

### 5.2 Correlation analysis of genetic risk factors and ASD

From Fig. 8 it can be seen that intron mutations accounted for a second high proportion of ASD-related genetic variations (24.4%). In all NS-CHD-related small variations (a total of 992 variation information), intron mutations ranked third of all the small variation types (20.5%). These data suggested that intron mutations play a pivotal role in the occurrence of CHD. It is generally assumed that the intron sequences do not play a role in pre-mRNA splicing process as it is far away from the classical splicing site. However, an increasing number of studies have shown that mutations in the intron region of many disease-related genes including single base mutations at the junction sites between introns and exons, can affect the splicing process of pre-mRNA. This alternative splicing often results in the generation of new exons in the mature mRNA product. It has been reported that in CHD and related complications, the c.3964 + 1G > T mutation in intron 32 of gene *FBN1* can contribute to Marfan syndrome [29]. Zhao et al. also found that the functional SNP mutation, c.56 + 781A > C, in the intron region of gene *MTRR* associated with the cysteine/folate metabolic pathway is an important genetic marker for ASD [30].

The diverse functions of introns, such as enhancement effects, promoter functions and other mediating factors can give introns more significant biological functions. The conservation of huge intron sequences in the human genome have special functions in biological evolution [31]. Therefore, more attention should be paid to intron mutations in genetic analysis. Based on the high percentage of intron mutations in ASD found in our database, the discovery and annotation of mutations in non-coding regions during analysis for CHD-related genetic variations should be a particular focus that can help to improve the diagnostic efficiency of genetic factors associated with CHD.

The GO annotation and the enriched GO terms at three major process levels are summarized in Table 2. At the BP level, the most frequently annotated gene was NRP1 (NM_003873.6) which had a total of 285 annotations. The most significantly enriched GO terms with target genes were mainly related to multiple cardiac septum development processes such as cardiac septum morphogenesis, outflow tract septum morphogenesis, ventricular chamber morphogenesis, and cardiac septum morphogenesis (Fig. 9A).

Comparative transcriptomics analysis demonstrated that cardiac-specific transcriptional factors (GATA4 and NKX2-5, which were annotated in our correlation analysis with ASD), extracellular signal molecules, along with cardiac sarcomeric proteins were downregulated in ASD. These changes may influence the formation of the heart atrial septum, cardiomyocyte proliferation, and cardiac muscle development [32]. The study also showed that the decreased expression of cell cycle proteins may affect cardiomyocyte growth and differentiation during atrial septum formation. At the MF level, the most annotated gene was NOS3 (NM_000603.5). A study on the role of NOS3 on myocardial performance indicated that NOS3 contributes to the bioactive NO pool during the development of sepsis and results in impaired cardiac contractility [33].

The most significantly enriched GO terms were mapped to NADP binding, oxidoreductase activity, acting on NAD(P)H, coenzyme binding, and flavin adenine dinucleotide binding. Compared to the enriched terms of isolated NS-CHD in the CHDGKB version [13], the enriched terms associated with ASD focused on sequence-specific DNA binding, DNA-binding transcription activator activity, enhance binding, and RNA polymerase II transcription factor binding (Fig. 9B). Previously, it has been shown that specific NKX2-5 mutations result in abnormal protein degradation through the Ubiquitin-Proteasome system and can contribute to CHD due to partially impaired transcriptional activity [34]. Furthermore, enhancers regulate transcription by binding to transcription factors which in turn could recruit cofactors to activate RNA Polymerase II at core promoters [35]. These changes demonstrate interactions between the processes of the ASD-related enriched terms described above. At the CC level, although the GO terms were not significantly enriched, the most frequently annotated gene was also NRP1 (NM_003873.6) which is the vital gene involved in the process of intermediate filament cytoskeleton that is a key receptor in the outflow tract of the developing heart septum [36]. Amongst the three process levels, the NOS3 gene is annotated in all three processes of the GO terms.

Based on the target genes related to ASD, we performed enrichment analysis of KEGG pathways and annotated a total of 107 KEGG metabolic pathways. These included the cGMP-PKG signaling pathway, fluid shear stress, atherosclerosis and cellular senescence. The significantly enriched pathways were mainly correlated with the cGMP-PKG and AGE-RAGE signaling pathway in diabetic complications (Fig. 9C). In the cGMP-PKG signaling pathway, four genes (GATA4, NOS3, EDNRA, and NFATC1) were found to be associated with ASD. These genes included GATA4, a zinc finger transcription factor that is essential for heart development and disease onset [37]. Other studies have shown that the transcriptional activity of GATA4 is mediated by cell signals that are dependent on cGMP- PKG-1α activity.

Protein kinase G (PKG) is a serine/tyrosine specific kinase and the main effector of cGMP signal transduction. Enhanced transcriptional activity induced by the co-expression of GATA4 and PKG-1α was also been observed. Phosphorylate GATA4 (S261) can be detected on Serine 261, and the C-terminal activation domain of GATA4 is related to PKG-1α. PKG-1α enhances the DNA binding activity of GATA4 through phosphorylation and physical connection processes. Many GATA4 mutations are associated with human diseases and exhibit impaired phosphorylation on S261 indicating that S261 phosphorylation defects are involved in human heart diseases [38], [39]. In summary, cGMP-PKG signaling mediates the transcriptional activity of GATA4 connecting GATA4 and PKG-1α mutations with human heart disease.

Another significantly enriched pathway involving ASD target genes was the AGE-RAGE signaling pathway which may be closely related to the influence of diabetes regulatory gene NOS3. Diabetes is also a risk factor related to CHD. It has been reported that NOS3, combined with TBX5 haploinsufficiency can cause abnormal heart formation [40]. These observations provide a new perspective on the molecular mechanisms of the combined impacts between genes, the environment, and other CHD risk factors.

## 6 Conclusions and future directions

Based on risk factor information that was correlated with the ASD subtype derived in our CHD-RF-KB, further correlation analysis was performed between other risk factors, complications, prognosis, and therapies for ASD. These data enabled the development of a prediction model for ASD diagnosis and prognosis using logistic regression [41] or other methods [42]. These applications could be extended to other NS-CHD subtypes to help users make precise assessments for the risk of NS-CHD onset, prognosis and inform diagnosis and treatment strategies.

The purposes and content domains of other existing congenital heart disease databases are largely different from CHD-RF-KB [43], [44] as our data was curated by original research in PubMed. However, CHDRFGB has several limitations. Firstly, transcriptional information was not included in the current database. We could include more CHD-associated functional variations aiming to determine the complex relationships between genes and regulatory networks. Secondly, data from clinical or animal studies could be used to validate the findings and to demonstrate the underlying mechanisms of multiple risk factors in the development of NS-CHD. Finally, as the scientific discovery paradigm shifted to a data-driven model [45], we will ensure that our knowledgebase is regularly updated and expanded to include new associations with environmental factors and integrate proteomic and epigenetic data, and artificial intelligence models of NS-CHD.

## 7 Data availability

CHD-RF-KB is freely available at http://www.sysbio.org.cn/CHDRFKB/.

## Author contributions

BS, LY and XL designed the research study. LY, XL, YC performed the literature searches, selected the studies and performed the data extraction. XL constructed the database. LY, and BS drafted the manuscript. BS conceived and supervised the work. All of the authors consented to all the data in the study, critically revised the manuscript and approved the final version.

### CRediT authorship contribution statement

**Lan Yang:** Methodology, Data curation, Writing – original draft, Writing-review, Investigation. **Xingyun Liu:** Data curation, Investigation, Software, Visualization, Writing-review. **Yalan Chen:** Data curation, Formal analysis, Resources. **Bairong Shen:** Conceptualization, Supervision, Writing-editing.

## Declaration of competing interest

The authors have no competing financial interests or personal relationships to declare that may influence the work reported in this paper.
